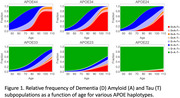# From Conflicting Data to Predictive Insights: A Digital Twin Approach for ADRD Risk and Resilience

**DOI:** 10.1002/alz70860_107177

**Published:** 2025-12-23

**Authors:** Jennifer Rohrs, Tom Paterson, Don Breuner, Cory C Funk

**Affiliations:** ^1^ Fulcrum Neuroscience, Palo Alto, CA, USA; ^2^ Institute for Systems Biology, Seattle, WA, USA

## Abstract

**Background:**

A vast amount of data has been collected on the genetic, lifestyle, and environmental risk factors contributing to Alzheimer's Disease and Related Dementias (ADRD); however, no consensus exists on how to integrate these findings for prediction of an individual's ADRD risk. Each study uses different inclusion/exclusion criteria, and variations in baseline age and follow‐up can significantly affect the results. For example, exercise is often cited as a key preventative measure for ADRD, yet its impact varies widely—showing strong effects in some studies (Larson, 2006), and no effect in others (Knutosor, 2021). Similar challenges exist for other risk factors, including diabetes, Vitamin D, and even the presence of amyloid plaques in cognitively normal individuals.

We have solved this challenge by analyzing ADRD risk and resilience data within the framework of a causal model, enabling us to reconcile seemingly disparate data and make individualized predictions using digital twins.

**Method:**

We developed a mechanistic model of brain health and neurodegeneration based on engineering principles of mass balance and closed‐loop feedback. The model was calibrated using in vitro, in vivo, clinical, and post‐mortem data. Biologically relevant variability was identified by examining mechanisms linked to hazard ratios. Using this model, we generated a digital population that replicates hazard ratio and Kaplan‐Meier data (Figure 1).

**Result:**

This digital population reconciles conflicting data and provides a consistent framework to compare the effect size of risk factors. We can stratify the population into subgroups based on which biological processes fail to compensate, leading to disease progression.

Additionally, we can create an individual's digital twin by using the digital population as a Bayesian prior then applying individual health data using Bayesian inference. The resulting digital twin can be used to predict the individual's risk of ADRD and guide personalized treatment and prevention strategies.

**Conclusion:**

Computational tools are needed to synthesize decades of ADRD research into causal hypotheses that accurately model the range of possible disease etiology. Mechanistic models, enhanced by artificial intelligence, offer an ideal framework for integrating diverse datasets and prior knowledge. Our scalable, high‐throughput platform enables the creation of digital twins, improving healthcare and optimizing treatment strategies.